# Surgical and oncological outcomes of laparoscopic right hemicolectomy (D3 + CME) for colon cancer: A prospective single-center cohort study

**DOI:** 10.1007/s00464-023-10095-w

**Published:** 2023-05-03

**Authors:** Xiaolin Wu, Yixin Tong, Daxing Xie, Haijie Li, Jie Shen, Jianping Gong

**Affiliations:** Department of Gastrointestinal Surgery, Tongji Hospital of Tongji Medical College, Huazhong University of Science and Technology, 1095 Jiefang Av, Wuhan, 430030 People’s Republic of China

**Keywords:** D3 + CME, Complete mesocolic excision, Right-sided colon cancer, Surgery, Survival

## Abstract

**Background:**

Complete mesocolic excision (CME) or D3 lymphadenectomy led to survival benefits for locally advanced right colon cancer, but with vague definitions in anatomy and debated surgical hazard in clinic. Aiming to achieve a precise definition of it in anatomy, we proposed laparoscopic right hemicolectomy (D3 + CME) as a novel procedure for colon cancer. However, the surgical and oncological results of this procedure in clinic were uncertain.

**Methods:**

We performed a cohort study involving prospective data collected from a single-center in China. Data from all patients who underwent right hemicolectomy between January 2014 and December 2018 were included. We compared the surgical and oncological outcomes between D3 + CME and conventional CME.

**Results:**

After implementation of exclusion criteria, a total of 442 patients were included. D3 + CME group performed better in lymph nodes harvested (25.0 [17.0, 33.8] vs. 18.0 [14.0, 25.0], *P* < 0.001) and the proportion of intraoperative blood loss ≥ 50 mL (31.7% vs. 51.8%, *P* < 0.001); no significant difference was observed in the complication rates between two groups. Kaplan–Meier analysis demonstrated that a better cumulative 5-year disease-free survival (91.3% vs. 82.2%, *P* = 0.026) and a better cumulative 5-year overall survival (95.2% vs. 86.1%, *P* = 0.012) were obtained in the D3 + CME group. Multivariate COX regression revealed that D3 + CME was an independent protective factor for disease-free survival (*P* = 0.026).

**Conclusion:**

D3 + CME could improve surgical and oncological outcomes simultaneously for right colon cancer compared to conventional CME. Large-scale randomized controlled trials were further required to confirm this conclusion, if possible.

**Graphical Abstract:**

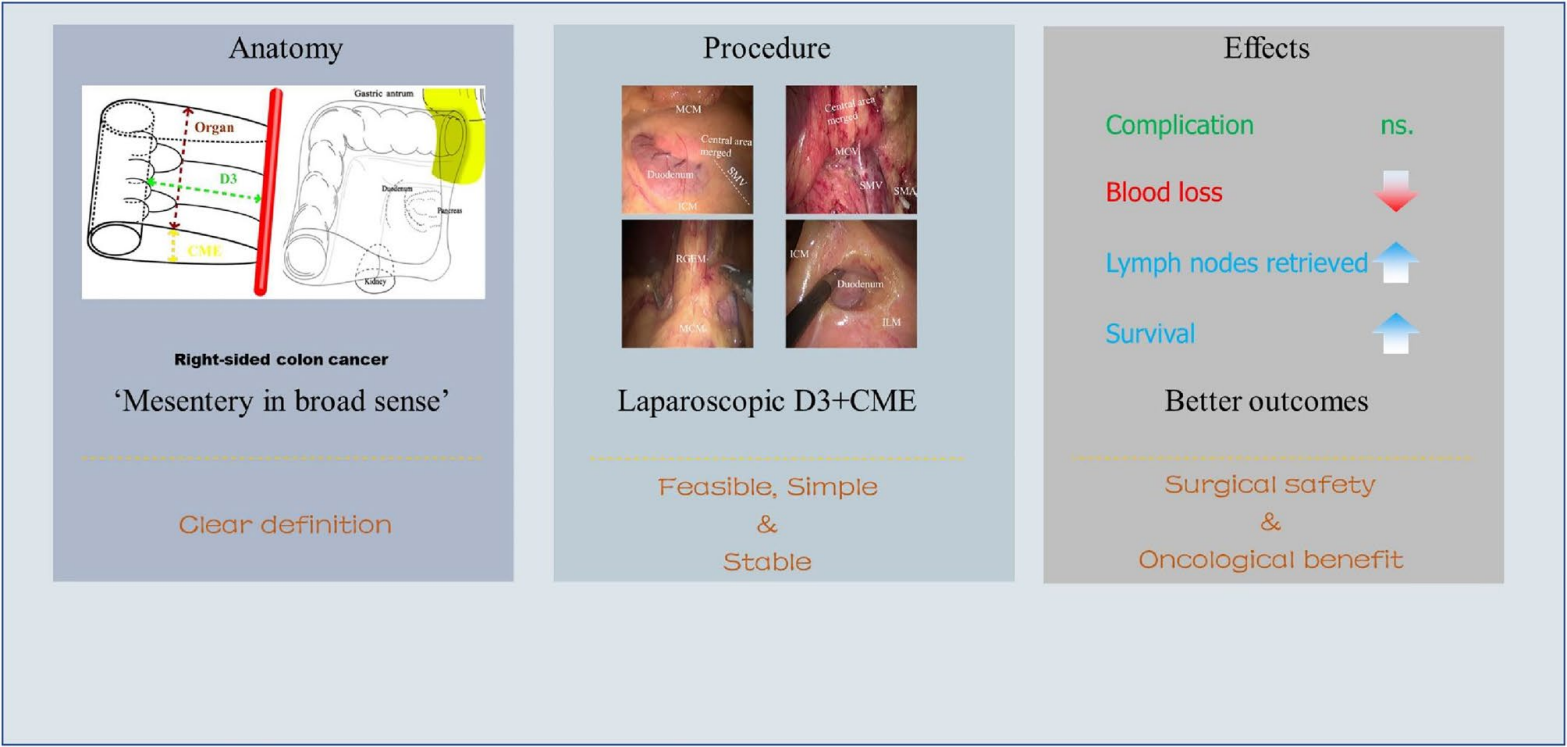

**Supplementary Information:**

The online version contains supplementary material available at 10.1007/s00464-023-10095-w.

Colorectal cancer is a threatening disease for human health worldwide, ranking third and second in terms of incidence and mortality among cancers, respectively [1]. Radical surgery remains the main treatment for locally advanced colorectal cancer [2].

Two surgical procedures have emerged in recent years: D3 lymphadenectomy advocated by Eastern countries [3] and complete mesocolic excision (CME) proposed by Western countries [4]. The former was defined as complete dissection of all regional lymph nodes (till D3 area) and transection of the colon 10 cm distal to the tumor [2], while the later emphasized the importance of removing mesocolon at the embryological planes and preserving the mesocolic integrity [4] (Figure S1). Both these two procedures seemed to be equally effective in improving the long-term prognosis of patients with colon cancer [5–11]. However, the surgical risks of both are controversial to date [6, 12–16]; the oncological benefits of D3 or CME surgery still lack high-quality evidence of randomized controlled trials [17–22]. In the paper published recently, “CME”, “CVL”, “D3” and other terms were used to describe or mark the surgical methods [7, 10, 23], but different studies were inconsistent as regard of procedure names and resection boundary [24]. The lack of a precise surgical definition based on anatomy may result in misinterpretation for the research results [25], which might be the major reason of various survival outcomes observed in different studies [26]. Briefly, it seemed that smeared-out definitions and debated surgical hazards were common characteristics of these procedures.

In order to make the resection boundary more precise, we proposed a novel procedure, i.e., laparoscopic right hemicolectomy (D3 + CME) [27], for colon cancer in 2017. Briefly, D3 + CME means complete excision of mesocolon in D3 boundary. In detail, right-sided mesocolon, as an envelop-like structure, is raised up from its bed to the root of the mesentery which is located at the bifurcation of branch vessels of superior mesenteric artery (SMA) and superior mesenteric vein (SMV). Then the branch vessels are ligated at their bifurcation, and the whole sample containing lymph nodes is removed as a bloc at last. In this procedure, “D3” means the distance from the primary lesion to the D3 area lymph nodes, “CME” means keeping the integrity of the mesentery as complete as possible. In this way, the procedure gets a definition with geometrical boundary (Fig. 1 and Figure S1) and ensures the integrity of mesentery as much as possible [28]. In recent years, live surgeries of D3 + CME have been conducted many times in Europe and China to show the feasibility of such a novel technique, and the replications of it have been completed by other centers. However, people wondered if such a procedure with a precise definition was safe or necessary. Here, we present the results of a prospective single-center cohort study from our team.

**Fig. 1:**
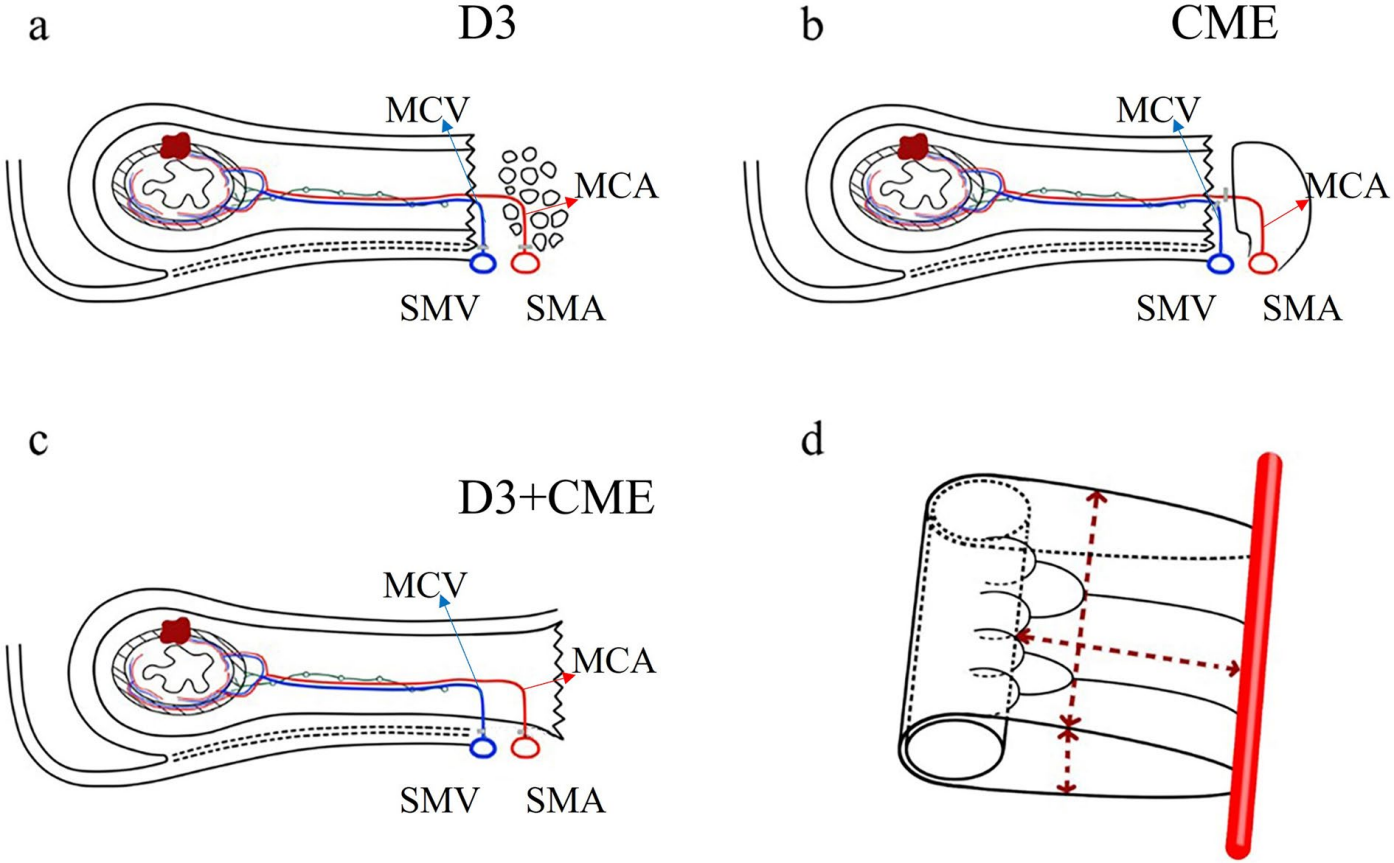
Schematic diagram of surgery in right-sided colon cancer. Schematic to show the procedure of **a** D3, **b** CME and **c** D3 + CME surgery, respectively. **d** Three-dimensional envelop-like structure of D3 + CME resection boundary. D3 + CME, D3 lymphadenectomy plus complete mesocolic excision; CME, complete mesocolic excision; D3, D3 lymphadenectomy; SMA, superior mesocolic artery; SMV, superior mesocolic vein. MCA, middle colic artery; MCV, middle colic vein. The brown parts indicated the tumor, the red parts indicated arteries, the bule parts indicated veins and the green parts indicated lymphatic system

## Methods

### Patients

This study included patients underwent right hemicolectomy from January 1, 2014 to December 31, 2018 in a single center (Department of Gastrointestinal Surgery, Tongji Hospital, Wuhan, China). Inclusion criteria: (1) Patients with right hemicolectomy; (2) Tumor sited in the caecum, ascending colon, hepatic flexure, or proximal transverse colon. Exclusion criteria: (1) Patients with emergency surgery or open surgery; (2) Patients with non-adenocarcinoma pathological results; (3) Patients with palliative resection or distant metastases. Information of patient demographic characteristics, preoperative assessment, intraoperative conditions, and postoperative outcomes was extracted in a prospective database. Preoperative anesthetic risk was assessed by American Society of Anesthesiologists (ASA) classification [29] for each patient. Charlson Comorbidity Index (CCI) [30] was calculated for each patient according to patient baseline information. Postoperative tumor pathological staging was performed according to American Joint Committee on Cancer (AJCC) 8th edition. All the participants provided informed written consent to participate in this study. Ethical approval for this study was granted by the hospital's ethics committee.

### Procedure

Laparoscopic D3 + CME surgery was performed by the inventor of this procedure in our center, and the surgical procedure was described in our previous research (attached with a surgery video) in detail [27]. Briefly, D3 + CME means that CME is made in D3 field, which requires the surgeon to identify three “Tri-junction” points and ensure the relative integrity of the mesentery when dissecting the fusion plane of different mesentery. Following this principle, the ileocolic mesentery and the middle colic mesentery, the right gastroepiploic mesentery and the middle colic mesentery, and the right mesocolon and its mesenteric bed are sequentially separated. The lateral boundary of D3 + CME is the fusion edge between the parietal peritoneum and the right mesocolon, the upper boundary is the fusion site of the right gastroepiploic mesentery and the middle colic mesentery, the lower boundary is the fusion site of the ileocolic mesentery and the ileum mesentery, the posterior boundary is the fusion plane of the right mesocolon and its mesentery bed (retroperitoneum, duodenum, pancreas, etc.), and the medial boundary is the left edge of the SMA. Figure S2 showed the sample of laparoscopic view of D3 + CME. While the procedures performed in the control group completed CME without following the D3 + CME concept, and was performed by other surgeons experienced in laparoscopic right hemicolectomy.

### Surgical outcome

Complications were graded by using the Clavien-Dindo classification [31] and included chyle leak, pulmonary infection, abdominal infection, wound infection, anastomotic fistula, intestinal obstruction, postoperative abdominal pain, and other complications. Intraoperative bleeding volume was estimated and recorded in the database by the anesthesiologist. Lymph nodes retrieving were performed by specialized pathologists.

### Patients Follow-up and Long-term Outcome

Follow-up data were registered prospectively in the medical records of the departments. In principle, follow-up was conducted three months interval for the first year after surgery, 6 months interval for the second year, and annually thereafter. The postoperative surveillance consisted of physical examination, quality of life, CT scan, blood tumor biomarker (including CEA, carbohydrate antigen (CA) 19–9 and CA72-(4), and the colonoscopy results. The primary long-term outcomes were cumulative disease-free survival (DFS) and overall survival (OS).

### Statistical analysis

Data normality was determined by the Kolmogorov–Smirnov test for continuous variables, with normally distributed data expressed as mean (*SD*) and non-normally distributed data expressed as median [*IQR*]; categorical variables were expressed as n (%). The t-test or Man–Whitney U test was used for continuous variables, and the chi-square test or Fisher exact test was used for categorical variables. Kaplan–Meier (KM) method and log-rank test was used for survival data analyzing and survival curves plotting. Univariate and multivariate COX regression were used to determine risk factors for disease-free survival. *P* value < 0.05 was considered statistically significant. Statistical analysis was performed using SPSS Statistics for Windows v26.0 (Armonk, NY: IBM Corp).

## Results

### Patients’ characteristics

**Fig. 2:**
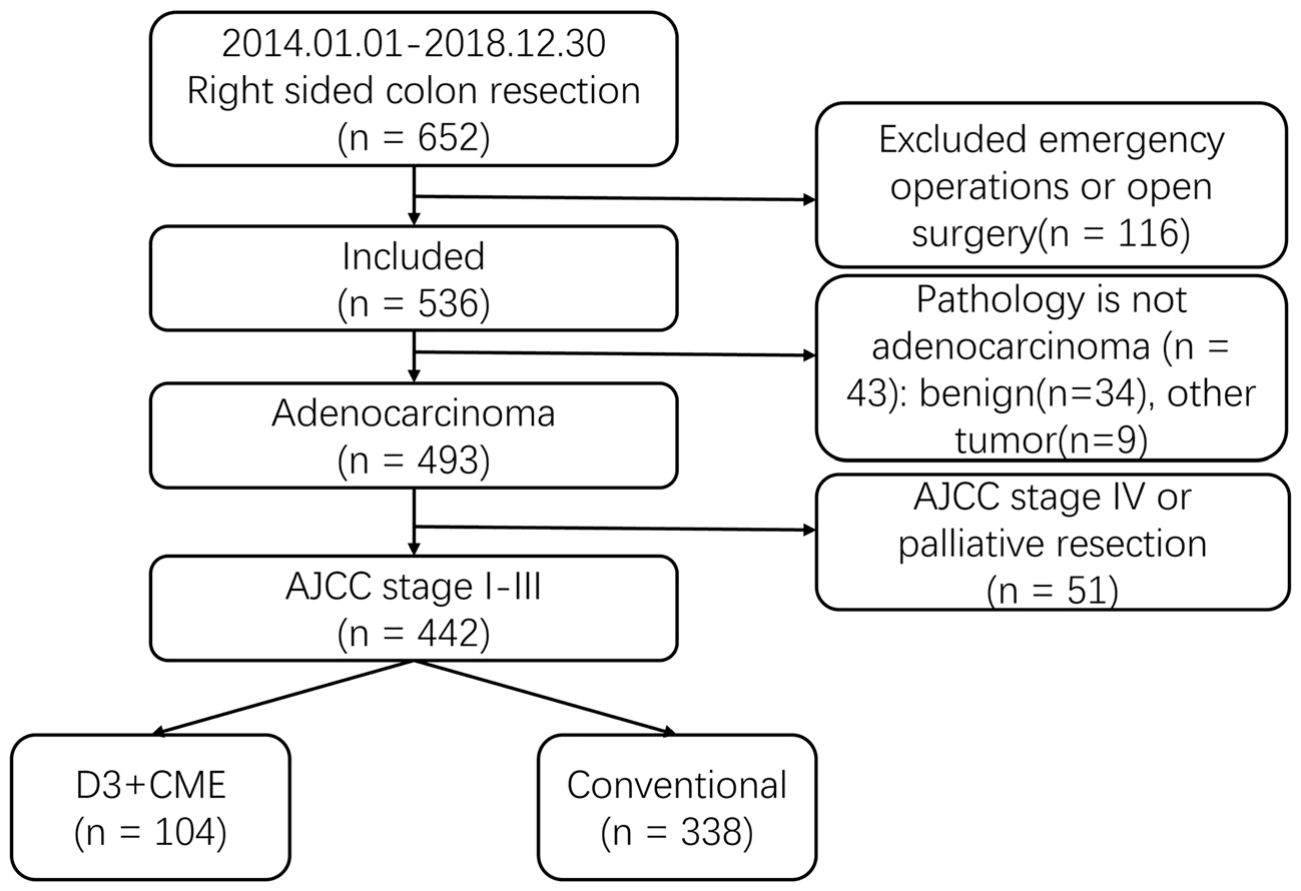
Patients selection flow chart

From January 1, 2014 to December 31, 2018, a total of 652 patients underwent right hemicolectomy were enrolled. One hundred and sixteen of them were excluded because they underwent emergency surgery or open surgery; forty-three patients with benign pathological findings and 9 patients with other tumors were excluded from the analysis; fifty-one patients with distant metastases or palliative resections were also excluded. The final patients included in the analysis were 104 patients in the D3 + CME group and 338 patients in the conventional group (Fig. 2).

The characteristics of the patients' baseline and pathology were shown in Table 1 and Table 2. All baseline characteristics as well as pathological results between the two groups were comparable.

**Table 1:** Baseline characteristics

Baseline characteristics	D3 + CME (*n *= 104)	Conventional (*n * = 338)	*P*
Male, *n * (%)	48 (46.2)	183 (54.1)	0.154
Age, mean (SD)	56.4 (13.5)	57.1 (13.3)	0.628
BMI, mean (SD)	22.3 (3.2)	21.9 (2.9)	0.238
Previous abdominal surgery, n (%)	35 (33.7)	91 (26.9)	0.184
Neoadjuvant therapy,*n * (%)	0 (0.0)	7 (2.1)	0.207
ASA score, *n * (%)			0.296
I	15 (14.4)	35 (10.4)	
II	78 (75.0)	277 (82.0)	
III	11 (10.6)	26 (7.7)	
CCI, median [IQR]	0.0 [0.0, 1.0]	0.0 [0.0, 1.0]	0.097
Tumor size (main axis), cm, median [IQR]	5.0 [4.0, 6.0]	4.0 [5.0, 6.0]	0.730
Tumor location, *n * (%)			0.516
Caecum	33 (31.7)	96 (28.4)	
Ascending colon	22 (21.2)	78 (23.1)	
Hepatic flexure	40 (38.5)	118 (34.9)	
Transverse colon (right sided)	9 (8.7)	46 (13.6)	

D3 + CME, D3 lymphadenectomy plus complete mesocolic excision; *SD* standard deviation; *IQR* interquartile range; *BMI* body mass index; *ASA* American society of anesthesiologists; *CCI* charlson comorbidity index; *P* < 0.05 was considered statistically significant

**Table 2:** Pathological results

Pathological results	D3 + CME (*n *= 104)	Conventional (*n* = 338)	*P*
pT stage, *n * (%)			0.183
1	2 (1.9)	11 (3.3)	
2	11 (10.6)	35 (10.4)	
3	73 (70.2)	202 (59.8)	
4a	18 (17.3)	80 (23.7)	
4b	0 (0.0)	10 (3.0)	
pN stage, n (%)			0.946
0	73 (70.2)	221 (65.4)	
1a	11 (10.6)	36 (10.7)	
1b	7 (6.7)	22 (6.5)	
1c	2 (1.9)	9 (2.7)	
2a	6 (5.8)	25 (7.4)	
2b	5 (4.8)	25 (7.4)	
Pathological stage, n (%)			0.106
I	9 (8.7)	40 (11.8)	
IIA	53 (51.0)	134 (39.6)	
IIB	11 (10.6)	41 (12.1)	
IIC	0 (0.0)	6 (1.8)	
IIIA	4 (3.8)	3 (0.9)	
IIIB	20 (19.2)	78 (23.1)	
IIIC	7 (6.7)	36 (10.7)	
Tumor differentiation, n (%)			0.931
Well	1 (1.0)	5 (1.5)	
Moderately	67 (64.4)	221 (65.4)	
Poorly	36 (34.6)	112 (33.1)	
R1 resection, n (%)	0 (0.0)	1 (0.3)	1.000

D3 + CME, D3 lymphadenectomy plus complete mesocolic excision; *P* < 0.05 was considered statistically significant

### Surgical outcomes

In the short-term outcomes (Table 3), the operative time was significantly longer in the D3 + CME group compared to the control group (256.0 [230.0, 288.0] vs. 236.5 [209.0, 262.3], *P* < 0.001). The proportion of patients with estimated intraoperative bleeding of more than 50 ml was significantly less in the D3 + CME group compared to the control group (31.7% vs. 51.8%, *P* < 0.001). There was also a significant increase in the number of lymph nodes retrieved in the D3 + CME group (25.0 [17.0, 33.8] vs. 18.0 [14.0, 25.0], *P* < 0.001). And in terms of hospital stay, there was a prolongation in the D3 + CME group (10.0 [9.0, 12.0] vs. 10.0 [9.0, 11.0], *P* = 0.026). No significant differences were found in the comparison of the conversion rate as well as the complication rate between the two groups.

**Table 3:** Surgical Outcome

Surgical outcome	D3 + CME (*n* = 104)	Conventional (*n* = 338)	*P*
Operation time, min, median [*IQR*]	256.0 [230.0, 288.0]	236.5 [209.0, 262.3]	< 0.001
Blood loss ≥ 50 ml, *n*(%)	33 (31.7)	175 (51.8)	< 0.001
Lymph nodes retrieved, median [*IQR*]	25.0 [17.0, 33.8]	18.0 [14.0, 25.0]	< 0.001
Lymph nodes involvement, median [*IQR*]	0.0 [0.0, 1.0]	0.0 [0.0, 1.0]	0.382
Conversion, *n*(%)	2 (1.9)	14 (4.1)	0.380
Complication			
Complications (*CD* = I), *n*(%)	0 (0.0)	9 (2.7)	0.124
Complications (*CD* = II), *n*(%)	10 (9.6)	42 (12.4)	0.491
Complications (*CD* ≥ III), *n*(%)	3 (2.9)	3 (0.9)	0.145
Abdominal infection, *n*(%)	1 (1.0)	2 (0.6)	0.554
Respiratory infection, *n*(%)	2 (1.9)	11 (3.3)	0.741
Abdominal pain, *n*(%)	1 (1.0)	4 (1.2)	1.000
Obstruction, *n*(%)	3 (2.9)	9 (2.7)	1.000
Leakage, *n*(%)	1 (1.0)	3 (0.9)	1.000
Bleeding, *n*(%)	0 (0.0)	4 (1.2)	0.577
Chyle leak, *n*(%)	1 (1.0)	2 (0.6)	0.554
Hospital stays, median [*IQR*]	10.0 [9.0, 12.0]	10.0 [9.0, 11.0]	0.026

D3 + CME, D3 lymphadenectomy plus complete mesocolic excision; *IQR* interquartile range; *CD* Clavien-Dindo classification; *P* < 0.05 was considered statistically significant

### Survival analysis

**Fig. 3:**
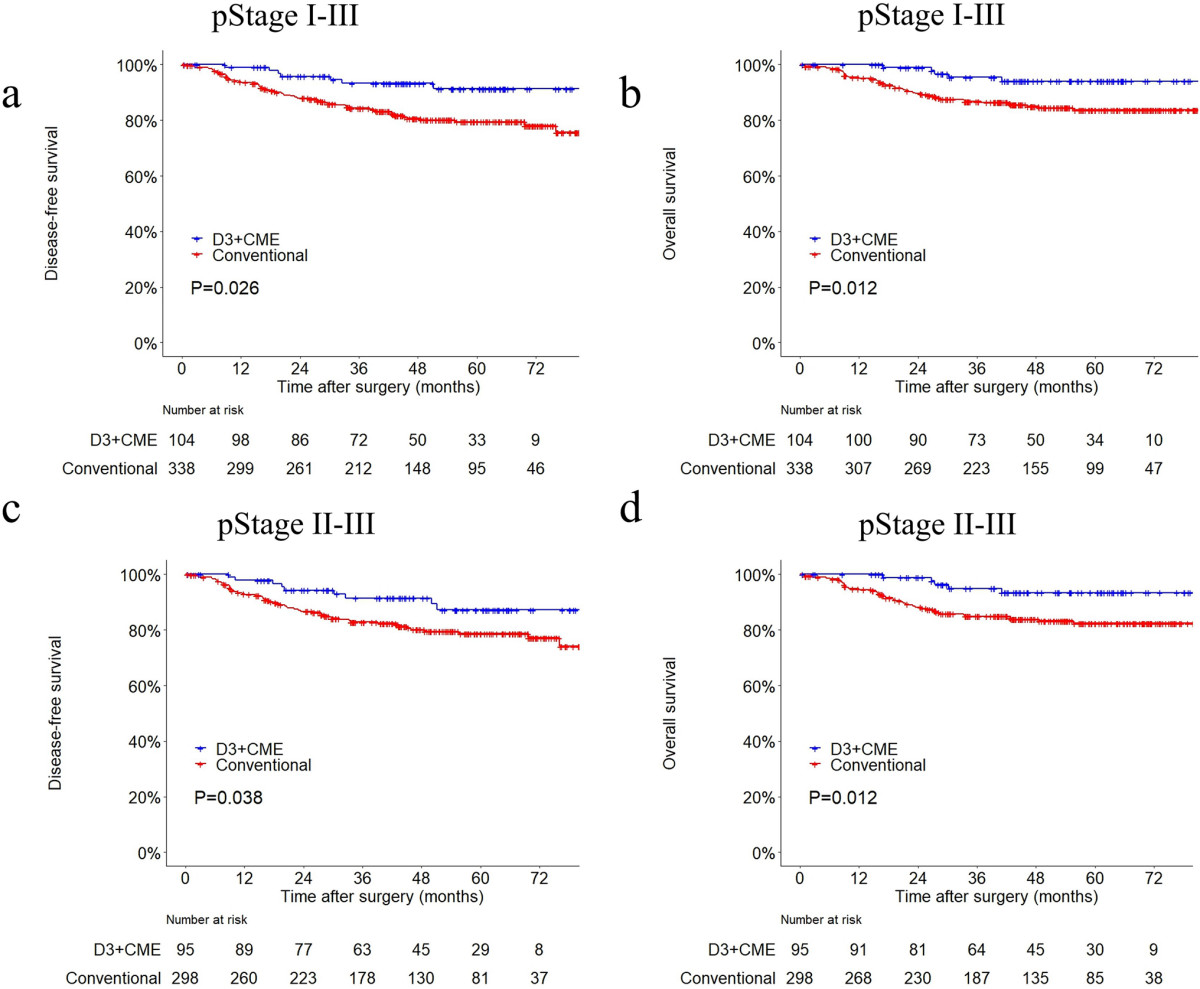
Kaplan–Meier curves for **a** disease-free survival and **b** overall survival in all cases; Kaplan–Meier curves for **c** disease-free survival and **d** overall survival in AJCC pathological stage II and III cases. **a** *P* = 0.026; **b** *P* = 0.012; **c** *P* = 0.038; **d** *P* = 0.012;

The median follow-up time was 46.5 (range 0.5–83.3) months for patients in D3 + CME group and 45.0 (range 0.4–85.7) months for the control group. The KM analysis (Fig. 3a, b) revealed that among all AJCC stage I-III patients, patients in the D3 + CME group had a better DFS than those in the control group (91.3% vs. 82.2%, *P* = 0.026), and the same findings applied to *OS* (95.2% vs. 86.1%, *P* = 0.012). In addition, after excluding patients with AJCC stage I (Fig. 3c, d), subgroup analysis showed that patients in the D3 + CME group still had a more favorable prognosis (DFS: 90.5% vs. 81.5%, *P* = 0.038 and *OS* 94.7% vs. 84.9%, *P* = 0.012, respectively). Stage I, II and III survival condition were demonstrated in Figure S3, respectively.

We identified independent factors associated with DFS by univariate and multivariate COX regression analysis (Table 4). Age (*P* = 0.025), pN stage (*P* < 0.001) and tumor differentiation classification (*P* = 0.028) were independent risk factors for DFS, while D3 + CME (*P* = 0.026) was an independent protective factor for DFS.

**Table 4:** Cox regression analysis of disease-free survival (backward stepwise likelihood ratio)

	Univariable analysis		Multivariable analysis	
	HR (95% *C.I*.)	*P*	HR (95% *C.I*.)	*P*
Age	1.017 (0.999–1.036)	0.063	1.021 (1.003–1.040)	0.025
Male	1.419 (0.874–2.302)	0.157		
CCI	1.138 (0.956–1.354)	0.147		
Tumor size	0.965 (0.838–1.112)	0.625		
Tumor location		0.655		
Caecum	Ref			
Ascending colon	0.983 (0.481–2.006)			
Hepatic flexure	1.359 (0.757–2.438)			
Transverse colon (right sided)	1.020 (0.443–2.346)			
pT stage		0.081		
pT1	Ref			
pT2	1.630 (0.196–13.543)			
pT3	1.965 (0.270–14.325)			
pT4	3.513 (0.476–25.933)			
pN stage		< 0.001		< 0.001
pN0	Ref		Ref	
pN1	3.080 (1.726–5.496)		3.118 (1.736–5.600)	
pN2	5.509 (3.128–9.703)		5.039 (2.834–8.961)	
Pathological stage		< 0.001		
I	Ref			
II	0.963 (0.364–2.542)			
III	3.876 (1.532–9.808)			
Tumor differentiation		0.020		0.028
Well	Ref		Ref	
Moderately	1.724 (0.236–12.608)		1.017 (0.135–7.646)	
Poorly	0.876 (0.120–6.393)		0.524 (0.070–3.911)	
Surgical procedure		0.030		0.026
D3 + CME	Ref		Ref	
Conventional	2.177 (1.080–4.387)		2.237 (1.103–4.537)	

D3 + CME, D3 lymphadenectomy plus complete mesocolic excision; *HR* hazard ratio; *C.I*. confidence interval; *CCI* charlson comorbidity index; *Ref*. reference; *P* < 0.05 was considered statistically significant

## Discussion

In this study, we expressed the resection boundary and key points of D3 + CME through geometrical boundary, avoiding a confusing concept to some extent. D3 + CME is not simple combination of D3 lymphadenectomy and CME, but integrates CME principle into the D3 lymph node dissection process [32], with clear three-dimensional geometric boundary described by resected colon length, distance from primary lesion to the roots of main branch vessels including D3 area lymph nodes, and envelop-like structure as peripheral boundary (Fig. 1d).

According to the results presented in our study, D3 + CME performed better in clinical outcomes: (1) Less surgical hazard: bleeding volume during operation reduced without complication rate increasing (Table 3). (2) Oncological benefit: the number of lymph nodes harvested increased, and 5-year cumulative *OS* and *DFS* improved (Fig. 3). Moreover, as a result of its clear definition, this procedure was of good feasibility and stability, as we have repeatedly demonstrated in domestic and international symposium by LIVE SURGERY; such an operation was also replicated by surgeons in other centers (personal communication), which suggested that it was fitted to popularization. In summary, the procedure, laparoscopic right hemicolectomy (D3 + CME), not only had a clearer geometric definition, but also could improve surgical and oncological outcomes simultaneously.

As we know, people have conducted trials to compare D3 surgery with D2 surgery [23] or CME with non-CME [26], but various conclusions about the oncological survival were drawn in different studies [25]. We compared the control group (CME) data in this study with the largest CME survival data that have ever reported [26], they seemed to be consistent with each other, which meant that the control group in this study was comparable and convincing (Table S1). As the results demonstrated, we compared the D3 + CME group with the control group of this study, and found that the survival of D3 + CME group was much better than conventional CME (Fig. 3).

Interestingly, data revealed that the survival advantage of D3 + CME also existed in those cases with pN0 stage (Figure S4). Such a result may not be completely explained by radical lymph nodes dissection. In previous studies, we proposed the hypothesis of “ Metastasis V” [33] which is different from the well-known direct invasion, lymphatic metastasis, hematogenous metastasis and implantation metastasis. Subsequently, we found that such a metastasis approach was independent of lymphatic metastasis in gastrointestinal cancer [34, 35], and preserving the integrity of the mesentery will minimize the scattered cancer cells in the mesenteric adipose tissue “leaking out” into the abdominal serous cavity [36]. This may explain, to some extent, the oncological advantages of D3 + CME in the cases without lymph node metastasis and provided another evidence supporting hypothesis of “Metastasis V”. Additionally, removal of occult positive lymph nodes which cannot be detected by conventional examination could also be a possible reason for this result.

We would like to propose an anatomical model here to explain the superiority of D3 + CME: “mesentery in broad sense”. Mesentery in broad sense (MBS) is an envelop-like structure that the proper facial membrane (and serous membrane in serous cavity) enclose the organ (or tissue) and its feeding structure (vascular system), suspending to and leading to the posterior wall of the body. MBS structures lie on (or are buried in) their mesenteric beds, and fuse with adjacent MBS structures or serous cavity wall. Moreover, MBS could be raised up to the root from its bed. Disruption of the envelop-like structure would lead to bleeding and cancer cell leaking, resulting in surgical risk and local recurrence.

There are four “weak” parts in MBS structure of the right mesocolon which were vague and imprecise, which could explain potential surgical hazard and local recurrence risk in conventional CME group: (1) The middle colic mesentery part is fused with the right gastroepiploic mesentery and can be regarded as a mesenteric bed for each other. In D3 + CME procedure, the middle colic mesentery could be stripped up from its bed (the right gastroepiploic mesentery) to the root [27, 37], avoiding any damage leading to bleeding or cancer cell leaking out from the envelop-like structure to the serous cavity. However, this anatomical feature had not been understood well and was difficult to identify in laparotomy, especially among surgeons specializing in colorectal surgery (Figure S2c). (2) The ileocolic mesentery is clustered. It could be easily raised up from its mesenteric bed, that is, the posterior wall of the serous cavity. In fact, there is a “thin” boundary between the ileocolic mesentery and the ileum mesentery, and the clustered ileocolic mesentery should be kept as complete as possible to avoid damage (Figure S2d). (3) The middle colic mesentery and ileocolic mesentery merge to form the “central part” of the right mesocolon, which can be stripped out from the anterior surface of SMV and SMA (Figure S2a and S2b). In this way, the central part of the right mesocolon can be removed completely without bleeding or adjacent structures (such as lacteal, visceral nerves and so on) damage, and more D3 area lymph nodes can be harvested, and the branches can be ligated at their bifurcation precisely. (4) In our opinion, the real envelop-like structure of the right mesocolon lies on the posterior wall of the serous cavity and fuses with it; the peritoneal “reflex” along the ascending colon is the only secondary “illusion” or the edge of the right mesocolon and its mesenteric bed. Inadequate understanding of these features or regarding the right mesocolon is “out of” the serous cavity would lead to wrong “plane anatomy” or envelop-like structure destruction. It is the MBS structure in which local life events occur that clarifies the boundary and definition of D3 + CME procedure.

In the framework of this geometrical model mentioned above, many phenomena and results seem to be explained well. Firstly, D3 + CME follows the geometrical boundary for surgical mobilization and resection, avoiding damage to adjacent structures during operation. Therefore, the seemingly expanded boundary of surgery would not increase the incidence of complications. Secondly, it keeps the integrity of the MBS structure as much as possible, protecting the blood vessels and lymphatic system in it from being damaged, and preventing scattered cancer cells from leaking out, resulting in less bleeding and better long-term outcomes. In addition, as a result of precise surgical boundary, D3 + CME is of good repeatability. In contrast, without clear understandings of the MBS structure and geometrical descriptions, conventional CME/D3 surgery might disrupt the MBS structure (destroying the integrity of the envelope or resulting in part of MBS structure residual) when mobilizing along the SMV and SMA, which would be a potential source of bleeding or cancer cell leaking (Table 3 and Fig. 3).

Based on the MBS model, we proposed an anatomical model named the proximal segment of dorsal mesogastrium (PSDM) in gastric cancer research [37], and subsequently proposed the MBS-based surgery named D2 + CME [38]; we then demonstrated the advantages of that procedure in surgical and oncological outcomes [36, 39–41]. The study D3 + CME for colon cancer here is amazingly consistent with D2 + CME for gastric cancer. This indicated that envelop-like structure of MBS model was of good compatibility to some extent. Surgical risk and oncological prognosis, as two major concerning problems that have puzzled surgeons for a long period, seemed to be solved in this geometric model by applying natural anatomic boundary. Based on the guidance of this model, we expected more and more envelop-like structures of MBS or “extra-bowel mesentery” would be identified, explored and utilized [42].

This current study suffered from a certain of limitations. Lower average BMI than western population, single-institutional design and relatively small sample size, which should be improved by well-designed randomized controlled trials in the following work.

In conclusion, laparoscopic right hemicolectomy (D3 + CME) with a more precise geometrical boundary and definition, seemed to be not only feasible, but also improving surgical hazard and oncological survival at same time.

## Supplementary Information

Below is the link to the electronic supplementary material.

Supplementary file1 (DOCX 3624 KB)
