# Correction to: Surgical and oncological outcomes of laparoscopic right hemicolectomy (D3 + CME) for colon cancer: A prospective single-center cohort study

**DOI:** 10.1007/s00464-023-10138-2

**Published:** 2023-05-18

**Authors:** Xiaolin Wu, Yixin Tong, Daxing Xie, Haijie Li, Jie Shen, Jianping Gong

**Affiliations:** Department of Gastrointestinal Surgery, Tongji Hospital of Tongji Medical College, Huazhong University of Science and Technology, 1095 Jiefang Av, Wuhan, 430030 People’s Republic of China

**Correction to: Surgical Endoscopy (2023)** https://doi.org/10.1007/s00464-023-10095-w

The original online version of this article was revised to correct errors in Fig. 3. In the original version of the figure, one sample was mistakenly grouped in the loaded data for the plotting of the Kaplan-Meire curves. In the risk table, there should be 104 people in the D3 + CME group instead of 103 people. Additionally, in the KM curves for Stage II-III, there should be 95 people in the D3 + CME group instead of 94 people.

**Fig. 3:**
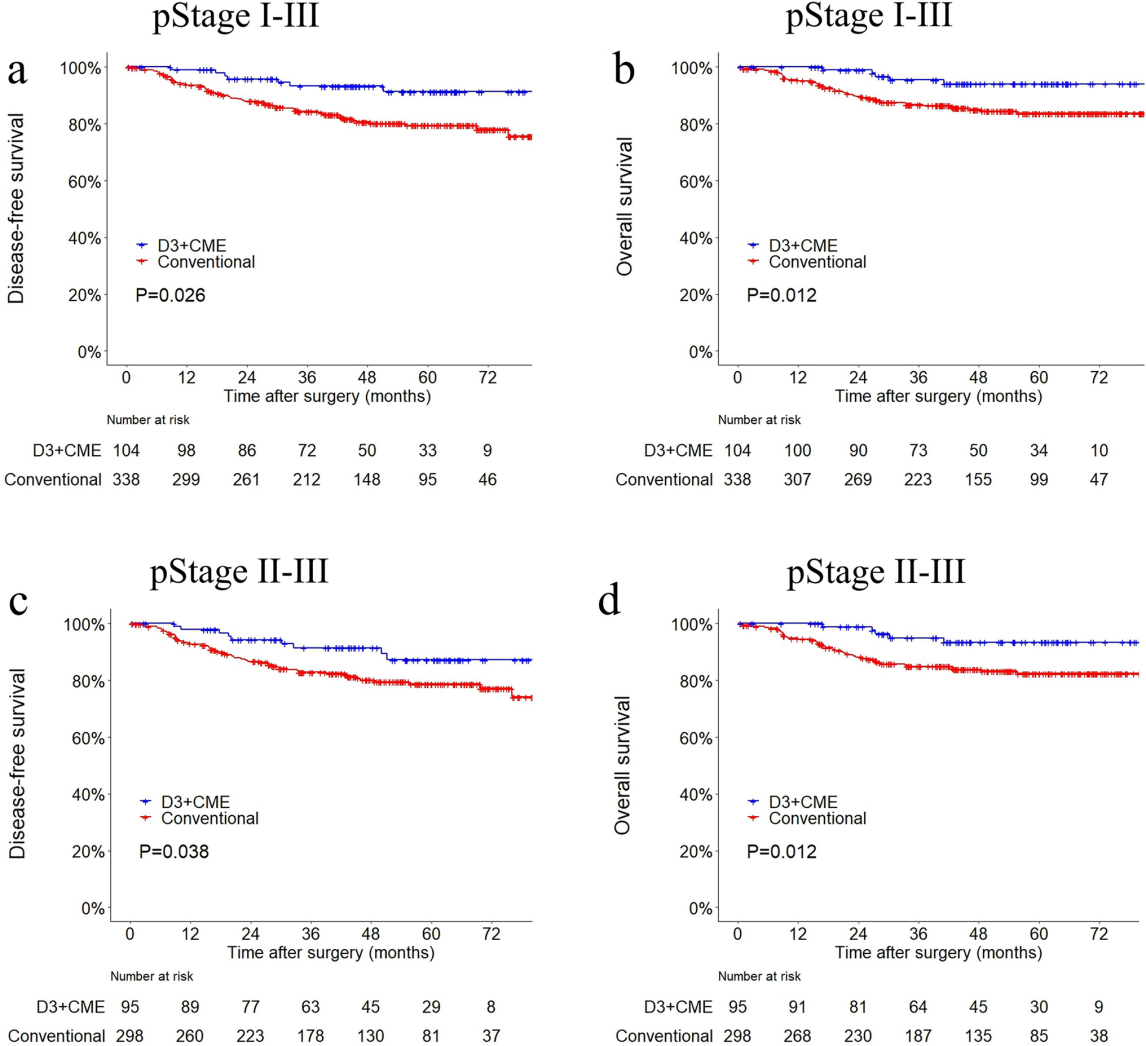
Kaplan–Meier curves for **a** disease-free survival and **b** overall survival in all cases; Kaplan–Meier curves for **c** disease-free survival and **d** overall survival in AJCC pathological stage II and III cases. **a** *P* = 0.026; **b** *P* = 0.012; **c** *P* = 0.038; **d** *P* = 0.012

The original article has been corrected.

