# Supplementary material for: Surgical and oncological outcomes of laparoscopic right hemicolectomy (D3 + CME) for colon cancer: A prospective single-center cohort study
Source: Surg Endosc. 2023 May 3;37(8):6107–17. doi: 10.1007/s00464-023-10095-w (PMC10338606; doi:10.1007/s00464-023-10095-w)
Supplement: Supplementary file 1 — Supplementary file1 (DOCX 3624 KB) [file 464_2023_10095_MOESM1_ESM.docx]

Supplementary Materials


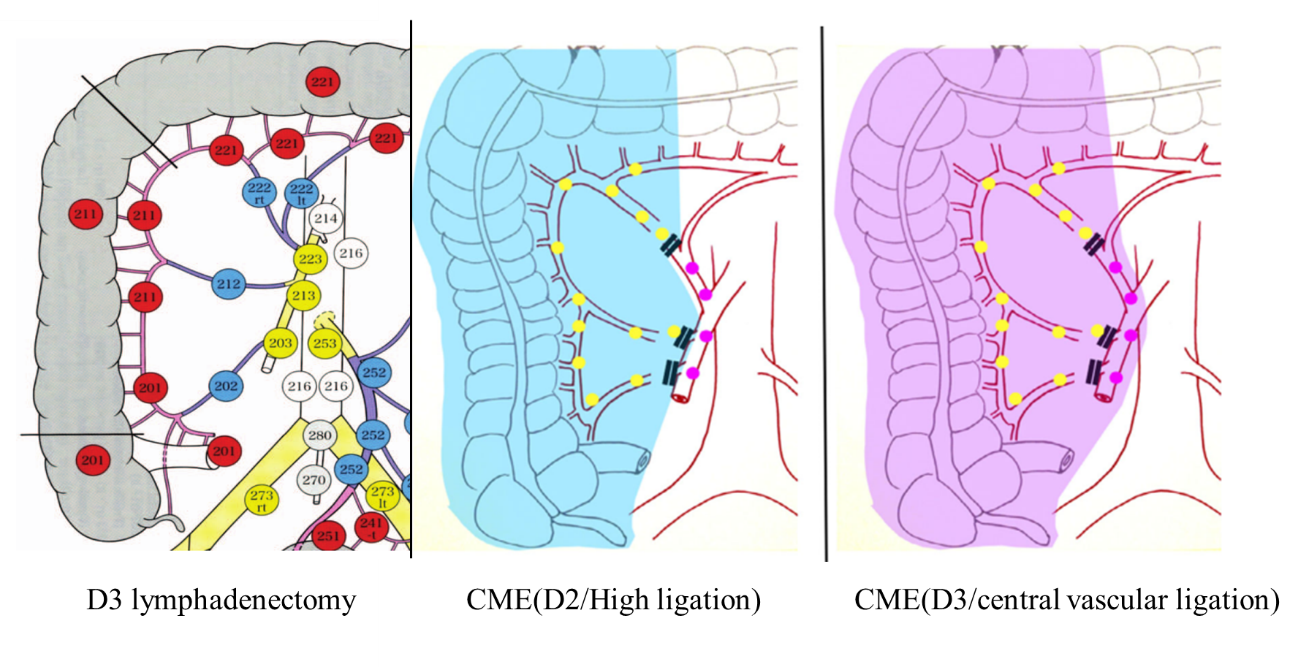


Figure S1. The schematic diagram for D3 lymphadenectomy, CME with high ligation and CME with central vascular ligation in the published literature^1,2^. CME, complete mesocolic excision.

**Reference**

1. Kataoka K, Beppu N, Shiozawa M, et al. Colorectal cancer treated by resection and extended lymphadenectomy: patterns of spread in left- and right-sided tumours. Br J Surg. Jul 2020;107(8):1070-1078.

2. Sammour T, Malakorn S, Thampy R, et al. Selective central vascular ligation (D3 lymphadenectomy) in patients undergoing minimally invasive complete mesocolic excision for colon cancer: optimizing the risk-benefit equation. Colorectal Dis. Jan 2020;22(1):53-61.


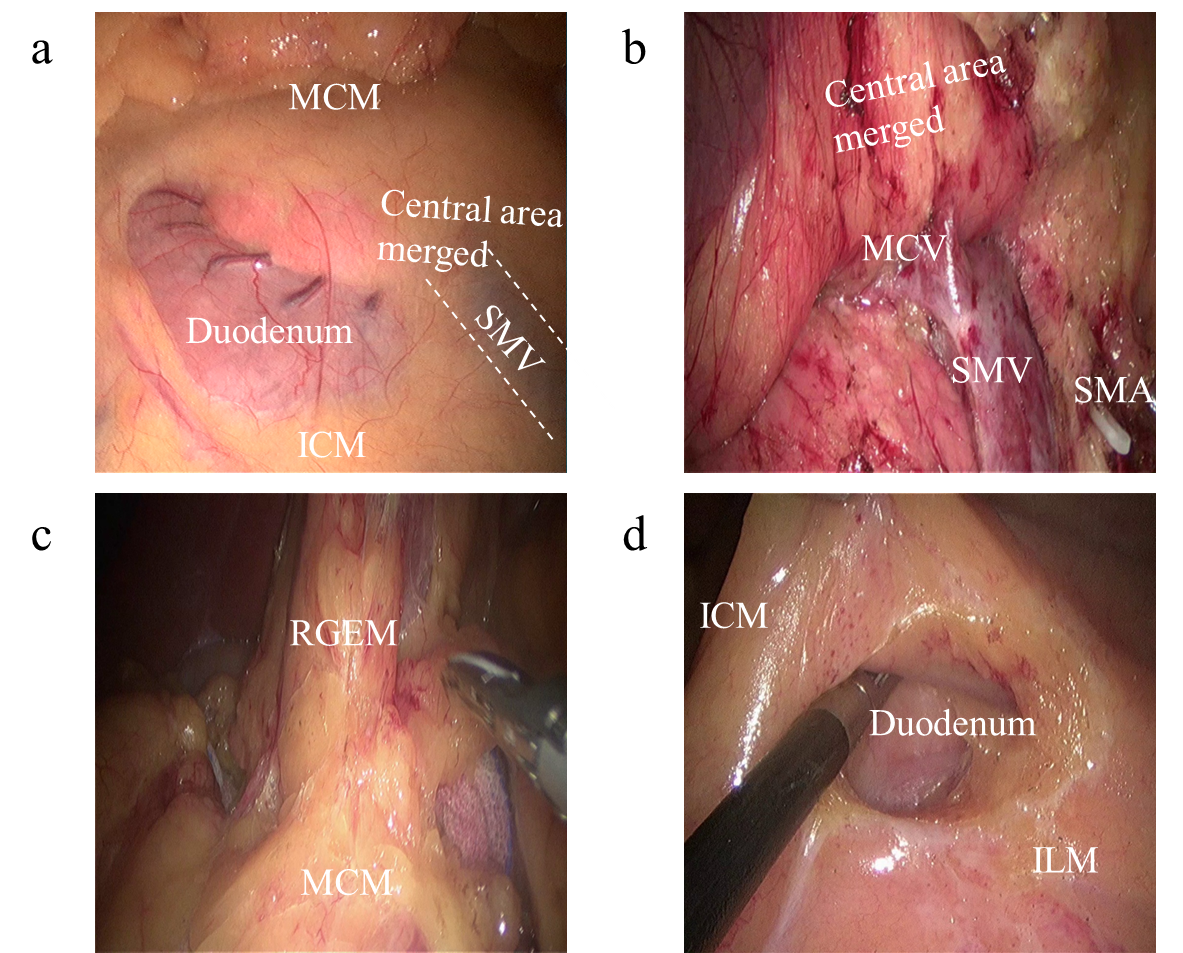


Figure S2. Intraoperatively laparoscopic view for right mesocolon boundary. **a.** Middle colic mesentery and ileocolic mesentery were merged to form the “central part” of the right mesocolon which is on the anterior side of SMV. **b.** The “central part” of the right mesocolon was stripped out completely to expose SMV and SMA. **c.** The merged area of right gastroepiploic mesentery and middle colic mesentery. **d.** The ileum mesentery and ileocolic mesentery were separated. MCM, middle colic mesentery; ICM, ileocolic mesentery; SMV, superior mesenteric vein; SMA, superior mesenteric artery; MCV, middle colic vein; RGEM, right gastroepiploic mesentery; ILM, ileum mesentery.


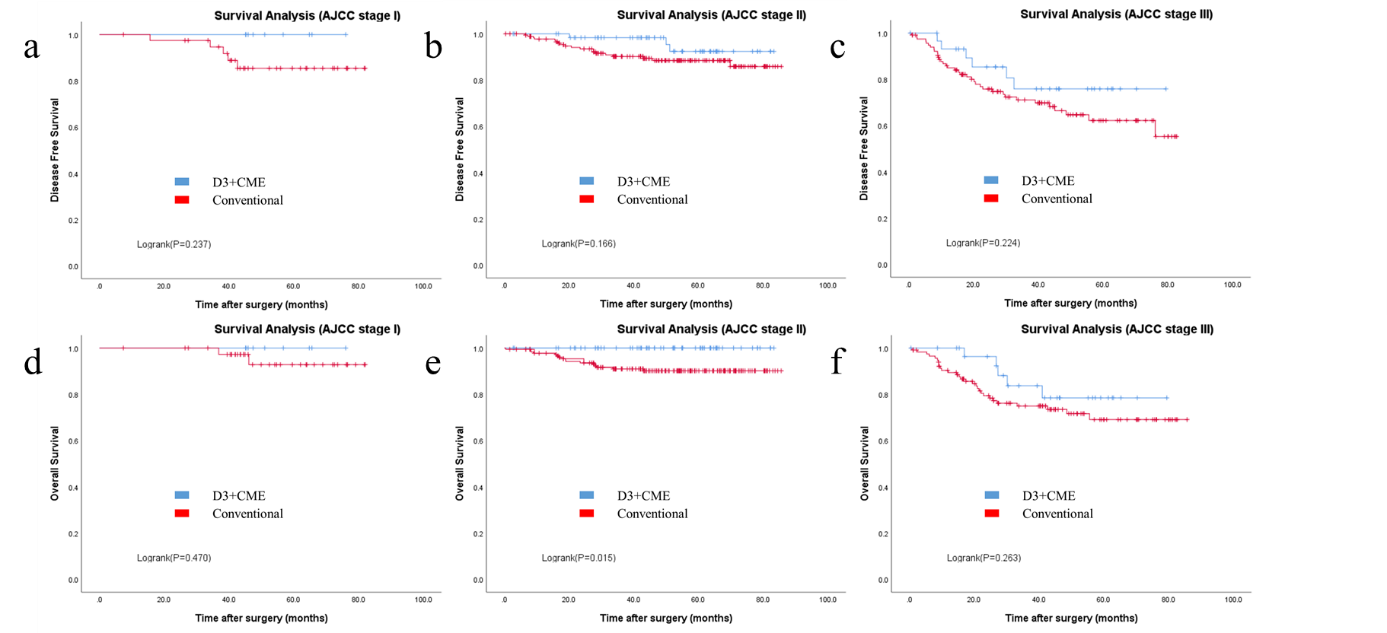


Figure S3. Comparison between D3+CME and conventional CME for disease-free survival of **a.** stage I, **b.** stage II and **c.** stage III, respectively. Comparison between D3+CME and conventional CME for overall survival of **d.** stage I, **e.** stage II and **f.** stage III, respectively.


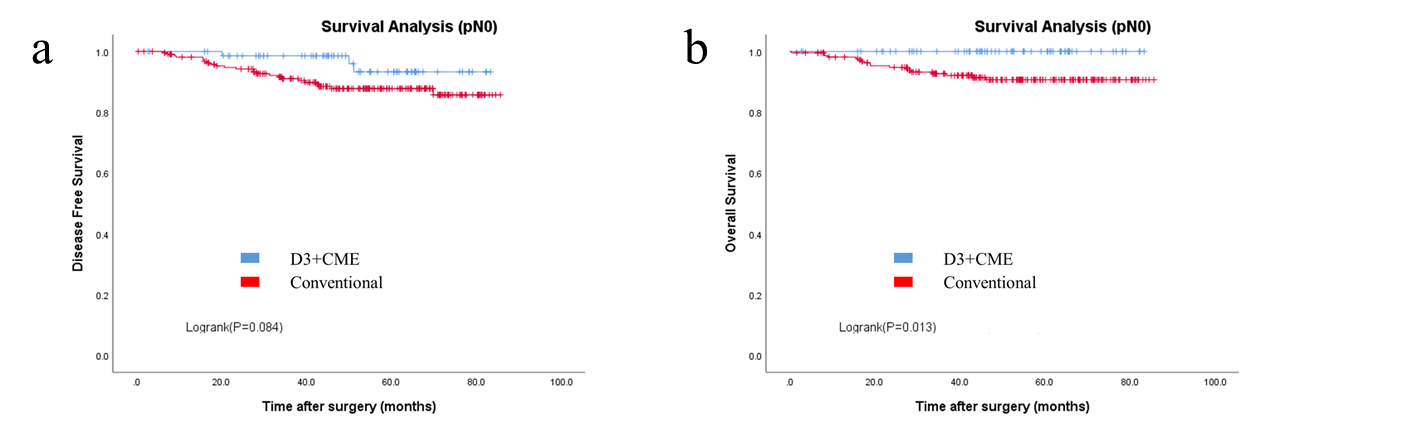


Figure S4. Comparison between D3+CME and conventional CME for **a.** disease-free survival and **b.** overall survival in pN0 cases.

Table S1. Overall survival comparison among the multi-center study conducted by Benz S. et al ^3^ and Storli K.E. et al ^4^, and this study, showing that the control group in our study was convincing.

|  | Cases, n | OS, % | Stage I, OS | Stage II, OS | Stage III, OS |
| --- | --- | --- | --- | --- | --- |
| Benz S. et al |  |  |  |  |  |
| CME | 496 | 81.4 | 83.2 | 83.8 | 78.3 |
| non-CME | 508 | 77.8 | 79.1 | 85.6 | 65.0 |
| Storli, K.E. et al |  |  |  |  |  |
| D2 | 105 | 79.0 | - | - | - |
| D3 | 84 | 88.1 | - | - | - |
| This study |  |  |  |  |  |
| Control | 338 | 86.1 | 95.0 | 91.2 | 75.2 |
| **D3+CME** | 104 | 95.2 | 100.0 | 100.0 | 83.9 |
| CME, complete mesocolic excision; D3+CME, D3 lymphadenectomy plus complete mesocolic excision; OS, overall survival | | | | | |

**Reference**

3. Benz, S.R., Feder, I.S., Vollmer, S., Tam, Y., Reinacher-Schick, A., Denz, R., Hohenberger, W., Lippert, H., Tannapfel, A., and Stricker, I. (2022). Complete mesocolic excision for right colonic cancer: prospective multicentre study. Br J Surg 110, 98-105. 10.1093/bjs/znac379.

4. Storli, K.E., Sondenaa, K., Furnes, B., Nesvik, I., Gudlaugsson, E., Bukholm, I., and Eide, G.E. (2014). Short term results of complete (D3) vs. standard (D2) mesenteric excision in colon cancer shows improved outcome of complete mesenteric excision in patients with TNM stages I-II. Tech Coloproctol *18*, 557-564. 10.1007/s10151-013-1100-1.
